# Managing failed vital pulp therapies in mature permanent teeth in a retrospective cohort study, with success and survival rates of managing protocols

**DOI:** 10.1038/s41598-024-62565-3

**Published:** 2024-05-21

**Authors:** Saeed Asgary, Leyla Roghanizadeh, Mohammad Jafar Eghbal, Alireza Akbarzadeh Baghban

**Affiliations:** 1 Iranian Centre for Endodontic Research, Research Institute of Dental Sciences, Shahid Beheshti University of Medical Sciences, Tehran, Iran; 2 Dental Research Center, Research Institute of Dental Sciences, Shahid Beheshti University of Medical Science, Tehran, Iran; 3 Proteomics Research Center, Department of Biostatistics, School of Allied Medical Sciences, Shahid Beheshti University of Medical Sciences, Tehran, Iran

**Keywords:** Medical research, Dental diseases

## Abstract

Despite advancements in vital pulp therapy (VPT), a subset of cases fails to achieve desired outcomes. This study based on a previous large-scale cohort study involving 1257 VPT-treated teeth, aiming to describe the demographic data and clinical characteristics of all failed cases and their management protocols. Clinical records/images of 105 failed cases treated by a single endodontist (2011–2022) were examined, including 10 extracted teeth. Asymptomatic cases with PDL widening received no intervention, while others underwent management protocols, including (selective) RCT and (tampon) re-VPT. These retreatments were assessed for success (defined as radiographic evidence of healing) and survival (characterized by the retention/function of the treated tooth) using Kaplan–Meier analysis. While 51.4% of all initial failures were diagnosed due to symptoms, 48.6% were symptom-free. Notably, failed cases with symptomatic irreversible pulpitis, and apical periodontitis/widened PDL before initial treatment significantly outnumbered asymptomatic cases and normal PDL, respectively (P = 0.001). Moreover, most of the initial failures were observed in teeth with composite resin rather than amalgam restorations (P = 0.002). The success and survival rates for the management protocols were 91.78% and 95.79%, respectively, over an average follow-up period of 36.94 (± 23.30) months. RCT and re-VPT procedures provide successful outcomes for managing unsuccessful VPTs.

## Introduction

Conservative treatment and minimal interventions are integral to modern dentistry, aiming to preserve dental tissues whenever possible^1^. In this regard, vital pulp therapy (VPT) holds significant importance in contemporary endodontic practice, particularly in preserving the vitality/function of the dental pulp in mature permanent teeth^2^. The American Association of Endodontists (AAE) emphasizes the significance of VPT, especially in cases involving irreversible pulpitis (IP) and carious pulp exposures, highlighting its advantages over traditional root canal therapy (RCT)^3^. Simple VPT techniques aim to conserve natural dentition by promoting healing and regeneration of dental pulp tissues, maintaining tooth structure, preserving occlusion, and mitigating potential complications associated with complex RCT procedures^4^.

Various VPT techniques, including direct/indirect pulp capping (DPC/IPC) and pulpotomy (miniature/partial/full; MP/PP/FP), are tailored to specific clinical scenarios^5^. DPC involves placing a biocompatible material directly over an exposed pulp to stimulate reparative dentin formation, effectively isolating and protecting the pulp from external irritants^6^. Pulpotomy, on the other hand, is indicated when the coronal pulp tissue is severely inflamed, while the radicular pulp exhibits lesser inflammation or is normal/healthy. This surgical procedure involves the removal of inflamed dental pulp tissue, ranging from minimal (MP) to the entire coronal pulp (FP), followed by applying a biomaterial to cap the pulpal wound. This process aids in promoting pulp vitality and healing^7^.

Research and meta-analyses have consistently reported high success rates ranging from 81 to 93% for different VPT procedures in long-term outcomes^6,8–10^. Moreover, studies comparing success rates of VPT with traditional RCT in mature permanent teeth demonstrate comparable outcomes, supporting VPT as a reliable alternative^11,12^. Calcium silicate-based cements, such as mineral trioxide aggregate (MTA) and calcium-enriched mixture (CEM) cement, have significantly advanced dentistry and demonstrated successful application in various endodontic and regenerative procedures, specifically VPTs^12^.

Despite generally favorable outcomes, a subset of VPT cases encounters treatment failure, necessitating detailed investigations into contributing factors^13,14^. Notably, post-treatment apical periodontitis (AP) radiographically signals VPT failure. The resorptive potential of granulation tissue and the potential destruction by infection byproducts present challenges in achieving healing for periapical lesions, which are globally characterized by a high incidence^15–17^.

This retrospective analysis, building on the foundation of the previous investigation^18^, aims to address the dearth of specific research on the causes of VPT failure in mature permanent teeth, identifying contributing reasons, evaluating management protocols, assessing survival rates, and investigating the impact of systemic conditions on VPT outcomes. The null hypothesis states that there is a significant association between the examined demographic data and clinical characteristics and the failure of VPTs in mature permanent teeth.

## Methods

The methodologies employed in the large-scale cohort study, extensively detailed in the original research^18^, were consistently the same in this continuation.

### Study design

This study systematically reviewed 105 cases of failed VPTs in mature permanent teeth, conducted by a single endodontist between 2011 and 2022. All cases analyzed were part of a previous large-scale cohort study that initially evaluated 1257 VPT cases^18^.

Data collection involved extracting information from electronic patient records and organizing it securely. Patient demographics, tooth characteristics, VPT techniques, treatment outcomes, management protocols, and follow-up duration were collected.

### Sample selection

Our methodology involved a retrospective analysis of patient records from the past decade to identify cases of failed VPT on mature permanent teeth. Inclusion criteria involved patients diagnosed with IP or AP, either presenting with symptoms or detected during follow-ups if they were asymptomatic. Additionally, if the treated tooth had been extracted, it was categorized as a failure case but was not included in the management protocol and further analysis. The characteristics of failed VPT cases were described, including tooth type, patient demographics, pre-treatment diagnoses, and treatment modalities. To assess the influence of various demographic data and clinical characteristics on VPT failure, we conducted statistical analyses to identify significant associations between patient, tooth, and treatment-related issues of VPT failure.

### Data collection

Data was collected from electronic patient records and organized securely and confidentially. The following information was extracted for each case:

1. *Patient demographics*: Age, gender, and relevant medical history, including the presence of systemic conditions such as diabetes mellitus, cardiovascular diseases, hyper/hypothyroidism, and medications received by the patient such as corticosteroids, bisphosphonates, cancer chemotherapy, etc.
2. *Tooth characteristics*: Tooth location, type of tooth, and preoperative pulp/periapical diagnosis.
3. *VPT technique*: Specific VPT technique employed (DPC, MP, or FP).
4. *Treatment outcomes*: Clinical and radiographic findings following initial VPT, including signs of treatment success or failure (such as persistent symptoms, or post-treatment PDL widening or AP).
5. *Management protocols*: The subsequent management approach that was employed after VPT failure including RCT, extraction, etc.
6. *Follow-up duration*: Length of follow-up period to assess outcomes.

### Radiographic examinations

Digital periapical radiographs were obtained using paralleling techniques and standardized exposure settings [X-ray unit: Soredex, Tuusula, Finland; XCP/Kit Rinn FPS 3000: Dentsply.

Sirona, India; Intraoral imaging plate system/Digora Optime: Soredex, Tuusula, Finland]^18^.

### Success/survival/event

In this study, success is defined as the absence of clinical signs/symptoms and radiographic evidence of healing, including the resolution of periapical lesions, after applying management protocols for failed VPTs. On the other hand, survival refers to the retention of the tooth in the oral cavity without the need for extraction after managing VPT failures. An event in our study context is defined as any occurrence that requires tooth extraction.

### Data analysis

Descriptive statistics were used to summarize patient demographics, tooth characteristics, VPT techniques, and treatment outcomes. The demographic data and clinical characteristics of failed VPT cases were analyzed using Cross-tabulations, and a Binomial test was applied to compare the distribution of failure reasons. Survival analysis was performed to assess the success and survival rates of different management protocols following VPT failure. Kaplan–Meier survival curves were generated, and log-rank tests were conducted to compare the survival rates between different retreatment modalities and no intervention. Data analysis was performed using SPSS software (Version 26.0; IBM Corp, Armonk, NY, USA), with a significance level set at P < 0.05.

### Ethics approval and consent to participate

The study adheres to ethical principles, with patient data treated confidentially and in compliance with data protection regulations. Ethical approval was obtained from the Ethics Committee of the Research Institute for Dental Sciences, Shahid Beheshti University of Medical Sciences (IR.SBMU.DRC.REC.1401.116). The need for informed consent was waived for the retrospective analysis of de-identified patient records, as approved by the aforementioned Ethics Committee. All methods were performed in accordance with ethical standards and research protocols.

## Results

Out of the 105 teeth diagnosed with unsuccessful VPT, Table 1 illustrates the various demographic data and clinical characteristics related to tooth, treatment, and failure in these cases. After excluding ten extracted teeth, the remaining cases (n = 95) were either left untreated if they were asymptomatic and the patient opted not to undergo retreatment (n = 22) or they underwent one of the management protocols (n = 73) and were subsequently analyzed after follow-ups.

**Table 1:** Demographic and clinical characteristics of 105 failed vital pulp therapy cases.

Variable	Frequency	Percent
Tooth type		
Incisors		
Upper	2	1.90
Lower	0	0
Premolars		
Upper	20	19.05
Lower	19	18.09
Molars		
Upper	24	22.86
Lower	40	38.09
Preoperative diagnoses		
Presence of preoperative restoration	54	51.40
Absence of preoperative restoration	51	48.60
Presence of symptomatic irreversible pulpitis	77	73.30
Absence of symptomatic irreversible pulpitis	28	26.70
Presence of PDL widening	34	32.40
Absence of PDL widening	71	67.60
Presence of apical periodontitis	36	34.30
Absence of apical periodontitis	69	65.70
Pulp therapy technique		
Direct pulp capping	62	59.00
Miniature pulpotomy	15	14.30
Full pulpotomy	28	26.70
Hemostasis (in ~ 2 min)		
Not achieved	35	33.30
Achieved	70	66.70
Restoration material type		
Amalgam	36	34.30
Composite resin	69	65.70
Restoration surfaces		
One	3	2.90
Two	52	49.50
Three	42	40.00
Four	8	7.60
Presence of symptoms at failure time		
Symptomatic failure	54	51.43
Asymptomatic failure	51*	48.57
Dentinal bridge formation (radiographically)		
Yes	10	9.50
No	95	90.5
Pulp canal calcification		
Yes	1	0.95
No	104	90.05

*PDL* periodontal ligament.

*Including 10 extracted teeth.

### Distribution of failed VPT-treated teeth

The distribution of failed VPT-treated teeth was as follows: 2 anterior teeth (1.90%), 39 premolars (37.14%), and 64 molars (60.95%). Notably, mandibular first molars comprised 23.81% (25 teeth) of the failed cases, followed by mandibular second premolars (18.09%, 19 teeth) and maxillary second premolars (14.28%, 15 teeth).

### Patient characteristics

The unsuccessful VPT-treated teeth belonged to 99 patients with a mean age of 43.34 ± 12.05 years. Out of the patients, 37 (37.4%) were male and 62 (62.60%) were female. The mean follow-up time from the time of initial VPT to the time of failure diagnosis was 36.94 ± 23.30 months, ranging from ≤ 1 to 9 years. Notably, some cases experienced failure within a short timeframe after VPT, as early as one month, indicating the need for prompt reassessment and implementation of alternative management strategies.

### Medical history and systemic conditions

Out of the total patient population, 78 were categorized as healthy non-smokers. Several failed teeth were associated with patients who presented underlying medical conditions. These included hypothyroidism (11 teeth, 10.48%), cardiovascular problems (10 teeth, 9.52%), type 2 diabetes (3 teeth, 2.86%), other conditions such as growth hormone deficiency, minor thalassemia, ulcerative colitis, cancer (with chemotherapy including long-term corticosteroids), multiple sclerosis (with long-term corticosteroids), rheumatoid arthritis (with long-term corticosteroids), joint arthrosis (with long-term corticosteroids) or osteoporosis (with oral bisphosphonate); each contributing to 1 tooth (0.95%). Additionally, seven failed teeth (6.70%) were from patients who smoked.

### Pre-treatment clinical diagnoses

The most common pre-treatment diagnosis was symptomatic IP, present in 77 teeth, significantly outnumbering asymptomatic cases (P = 0.001). Additionally, AP and widened PDL were observed in 70 teeth, significantly more than normal PDL cases (P = 0.001).

### VPT/restoration techniques and materials

The frequencies of different VPT techniques using CEM cement were as follows: DPC (62 teeth), FP (28 teeth), and MP (15 teeth). In 35 teeth hemostasis was not achieved within 2 min, prompting the application of the tampon approach^19^. This involved placing an endodontic biomaterial i.e. CEM cement into the cavity to facilitate hemostasis by applying mechanical pressure and promoting vasoconstriction to stop the excessive bleeding^20^.

The majority of failures occurred in teeth with composite resin restorations (69 teeth), rather than amalgam restorations (36 teeth), with a significant proportion (P = 0.002). In terms of the surface number of the coronal restoration, 50 teeth (47.62%) had three or more surfaces that were lost and restored.

### Failure diagnosis/reasons

Among the failed cases of initial VPT, 54 cases (51.43%) were diagnosed due to the occurrence of symptoms, while other cases were diagnosed incidentally during follow-up radiographs (48.57%). These failures manifested over various time intervals, spanning from ≤ 1 month to 9 years. Notably, 17.14% of these failures manifested symptoms within the first 6 months, with a cumulative total of 29.52% occurring within the first year following the initial VPT procedures. Moreover, 10 teeth and 1 tooth had radiographic signs of dentinal bridge formation and root canal calcification at the time of failure diagnosis, respectively. Among the identified failure reasons in the 95 cases analyzed, recurrent caries (6.70%), prosthodontic failure (4.80%), crown-root fracture (0.95%), and combined endodontic-periodontal disease (0.95%) were contributors.

### Management methods after VPT failure

**Figure 1:**
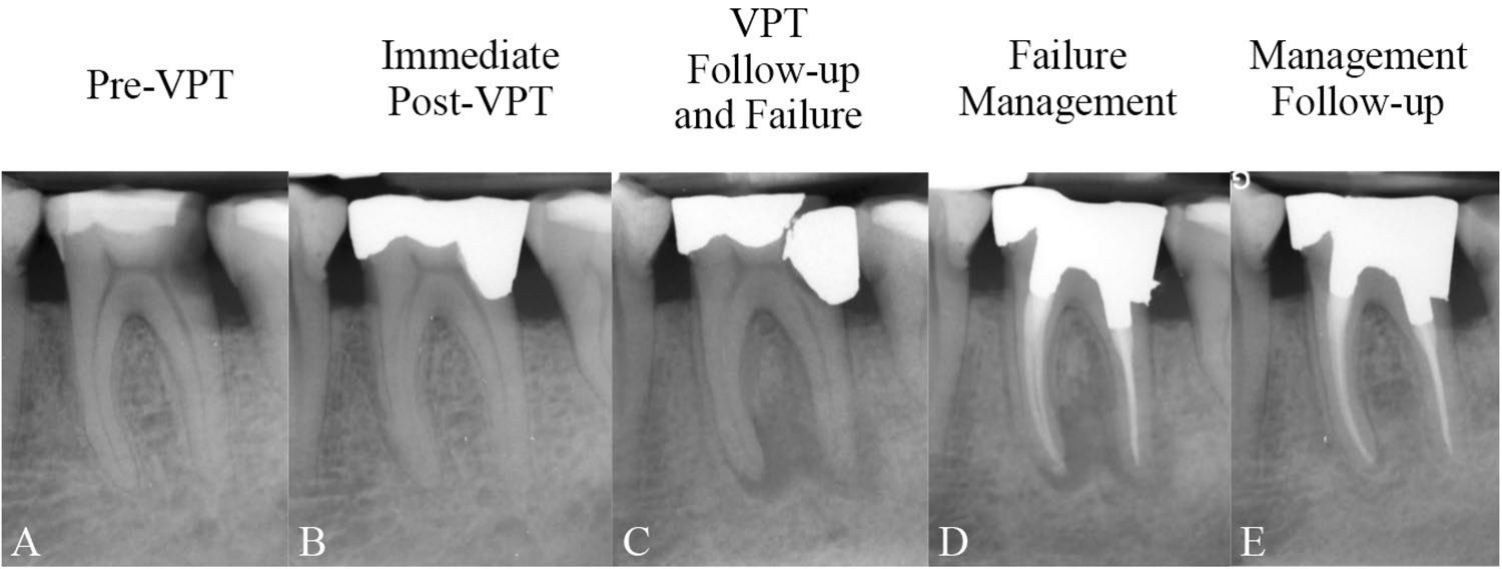
(**A**) Carious lesion on the lower left first molar of a 60-year-old man diagnosed with irreversible pulpitis; (**B**) Direct pulp capping and amalgam filling performed; (**C**) Fracture of the restoration and periapical periodontitis evident at 36-month follow-up, confirming failure; (**D**) Root canal treatment and amalgam build-up of the tooth completed in a single session; (**E**) Complete healing of the periapical lesion observed at 27-month follow-up, confirming success.

**Figure 2:**
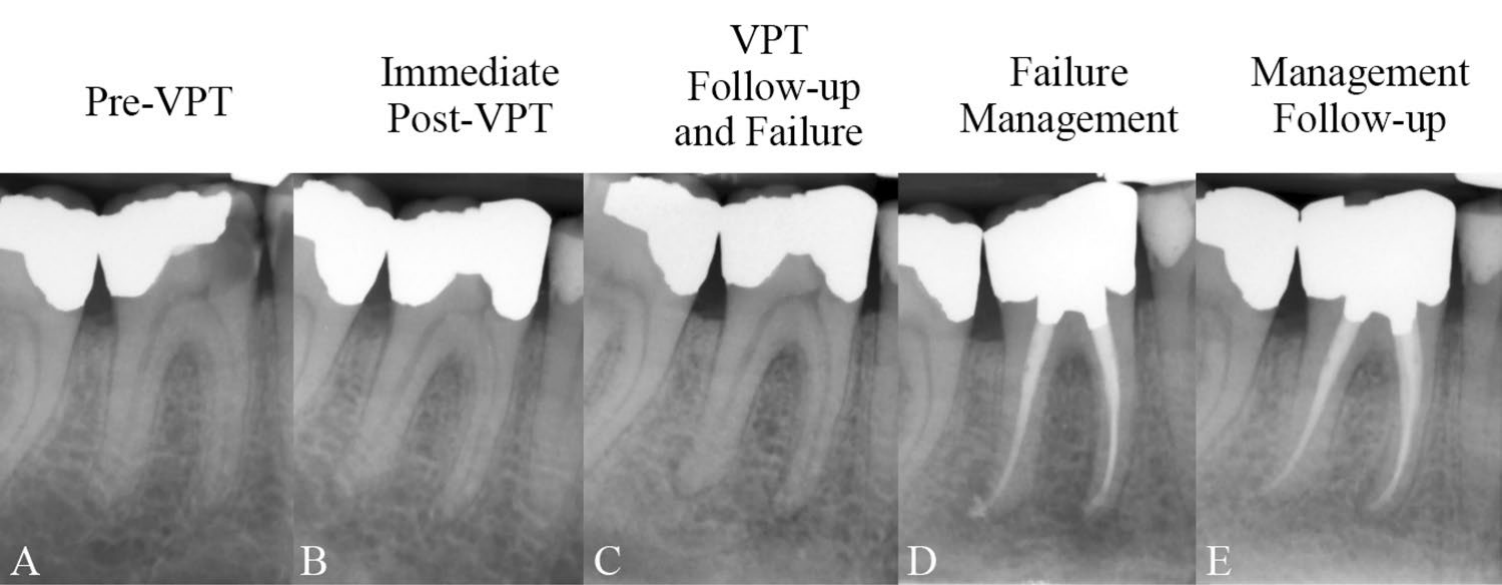
(**A**) Carious lesion on the lower right first molar of a 41-year-old man diagnosed with irreversible pulpitis; (**B**) Direct pulp capping and amalgam filling carried out; (**C**) Symptomatic tooth with periapical periodontitis evident at 6-month follow-up, confirming failure; (**D**) Root canal treatment and amalgam build-up of the tooth completed in a single session; (**E**) Healed periapical lesion and functional tooth identified at 44-month follow-up, confirming success.

**Figure 3:**
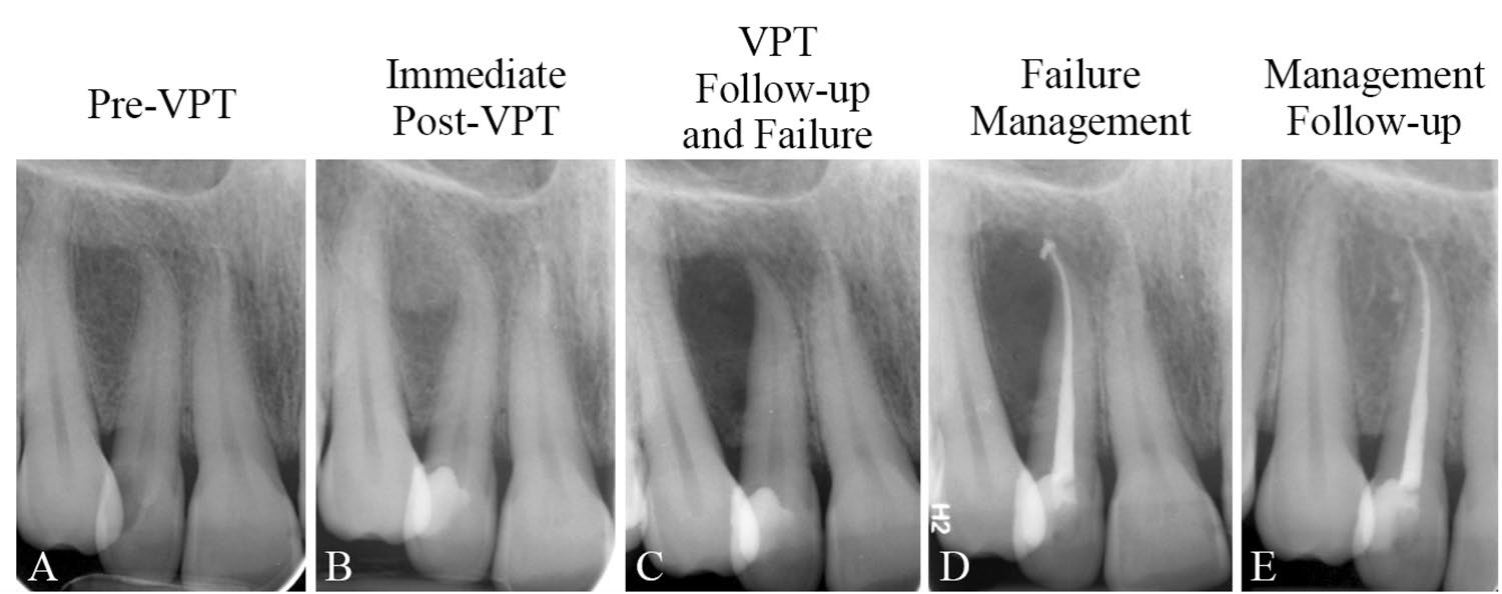
(**A**) Carious lesion on the left upper lateral incisor of a 37-year-old woman diagnosed with irreversible pulpitis; (**B**) Direct pulp capping and resin composite restoration performed; (**C**) Symptomatic apical abscess/large endodontic lesion detected at 13-month follow-up, confirming the failure; (**D**) Root canal treatment of the involved lateral incisor and filling/sealing of the access cavity using resin composite performed in a single session; (**E**) Complete healing of the large endodontic lesion noted at 34-month follow-up, confirming success.

**Figure 4:**
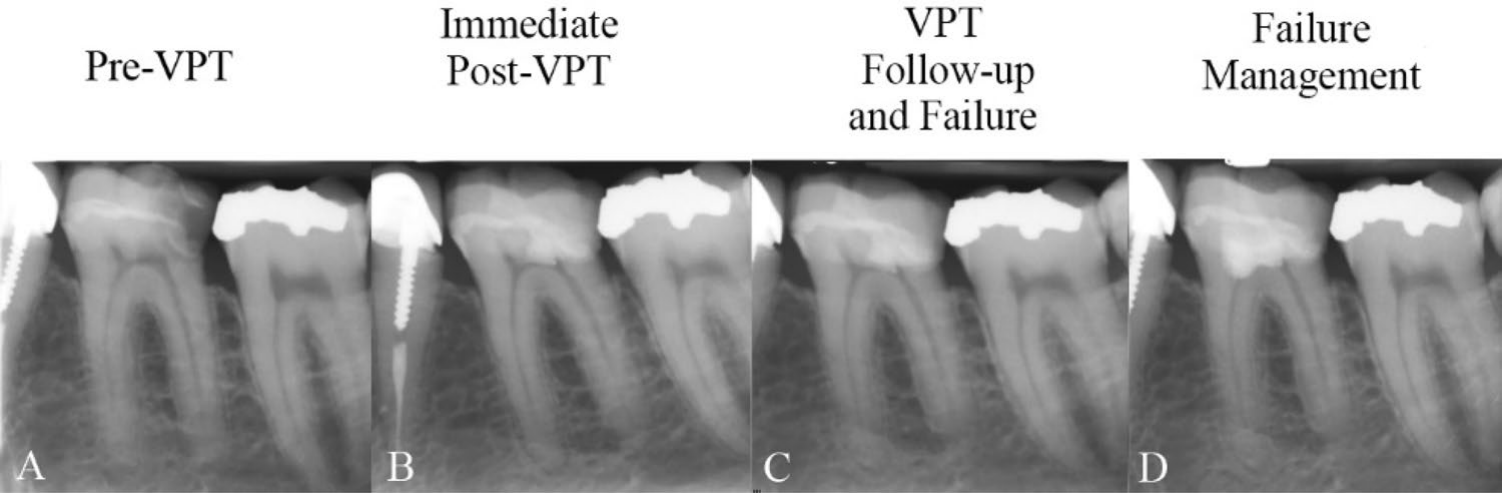
(**A**) Carious lesion on the lower left first molar of a 34-year-old woman diagnosed with irreversible pulpitis; (**B**) The affected tooth treated with partial pulpotomy and resin composite restoration. (**C**) Symptomatic clinical features of irreversible pulpitis associated with apical periodontitis observed at the 5-month follow-up, despite the absence of radiographic changes, indicating treatment failure; (**D**) Full pulpotomy (re-VPT) with a tampon approach (due to excessive bleeding) and resin composite restoration of the affected tooth performed; complete resolution of symptoms within one-week post-treatment, indicating success.

**Figure 5:**
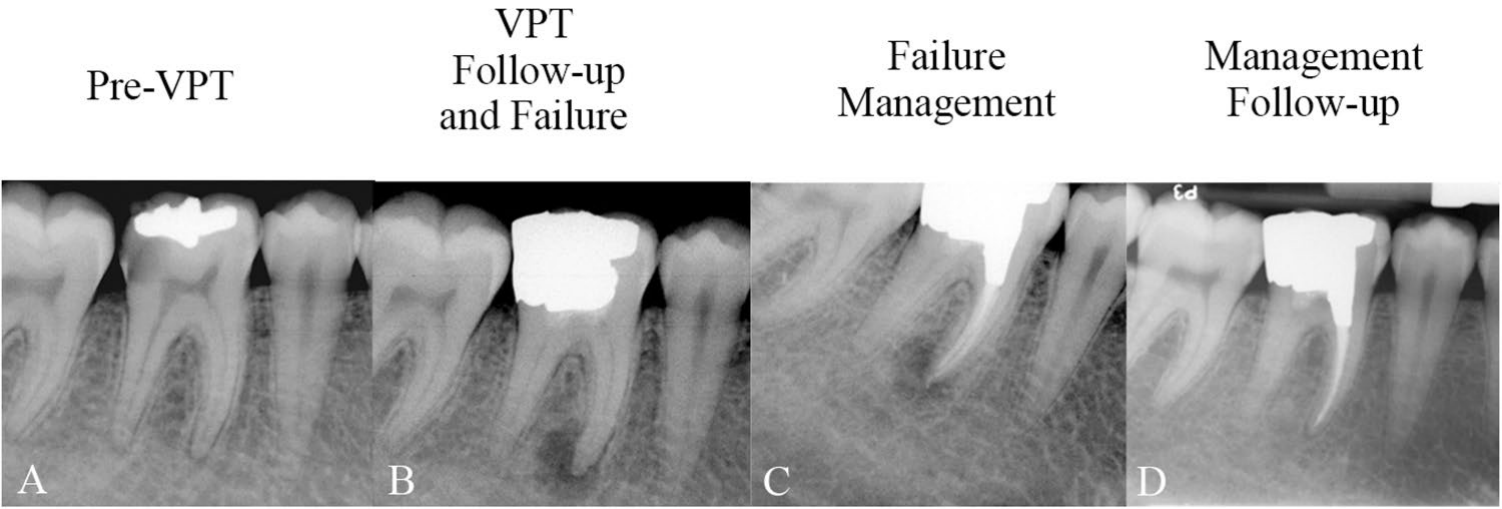
(**A**) Carious lesion on the right lower first molar of a 29-year-old man diagnosed with irreversible pulpitis/apical periodontitis, treated with full pulpotomy and amalgam filling; (**B**) Asymptomatic endodontic lesion detected during a periodic dental visit at 19-month follow-up, confirming failure; (**C**) Pulpectomy (selective RCT) and obturation of mesial canals and amalgam restoration of the involved tooth performed while the distal root remained untouched; (**D**) Complete healing of the periapical lesion noted at 42-month follow-up, with the tooth remaining functional, confirming success.

**Figure 6:**
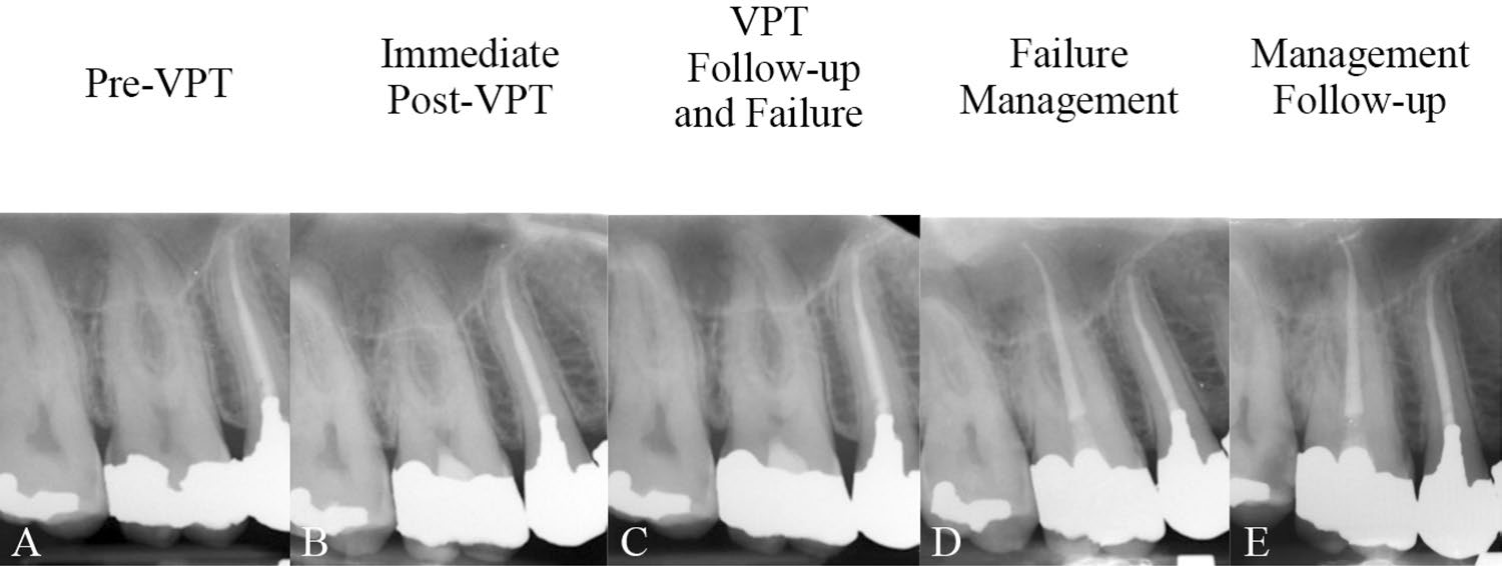
(**A**) The right upper first molar of a 26-year-old man, recently restored with amalgam, diagnosed with symptomatic irreversible pulpitis; (**B**) Pulpotomy and amalgam filling performed; (**C**) At 12-month follow-up, the symptomatic apical periodontist of the treated tooth was diagnosed, while a dental bridge formed beneath the capping biomaterial; (**D**) Pulpectomy (selective RCT) and obturation of the palatal canal (open orifice with necrotic tissue observed) and amalgam restoration of the tooth completed in a single session while the buccal roots remained untouched due to dentinal bridge formation at the canal orifices; (**E**) At 37-month follow-up the tooth was asymptomatic and functional, confirming success.

Table 2 shows the different management methods employed for the failed VPT cases. These methods include (selective) RCT for 69 teeth (Figs. 1, 2 and 3) and FP as a re-VPT for 4 teeth with/without tampon approach^19,20^ (Fig. 4). Where possible, the canal(s) causing the periapical lesion were identified and only those canal(s) were treated by RCT (Figs. 5, 6). Cases where teeth were asymptomatic and functional and the patient chose not to undergo re-treatment (22 teeth) were categorized as “no intervention”. Additionally, previous tooth extractions (10 teeth) were counted as failures but were not included in the success/survival analyses of management protocols.

**Table 2:** The different methods used for management of teeth after failed vital pulp therapy, and the rates of success and survival of each method.

Post-VPT failure management method	Frequency of management	Percent of management	Ultimate outcome				Final follow-up time of Management Method (Mean ± SD)
			Success frequency of the method	Success percent (rate) of the method itself	Survival frequency of the method itself	Survival rate of the method itself	
RCT	69	65.71	64	92.75	65	94.20	34.75 ± 22.36
FP	4	3.82	3	75.00	4	100	60.33 ± 36.69
EXT	10	9.52	NA	NA	NA	NA	NA
NI	22	20.95	NA	NA	22	10.00	43.60 ± 19.64
Total	105	100.0	67	91.78*	91	95.79^#^	36.94 ± 23.30

*RCT* root canal therapy, *FP* full pulpotomy, *EXT* extraction, *NI* no intervention, *NA* not applicable.

*Calculated as (67/73 * 100), ^#^Calculated as (91/95 * 100).

**Figure 7:**
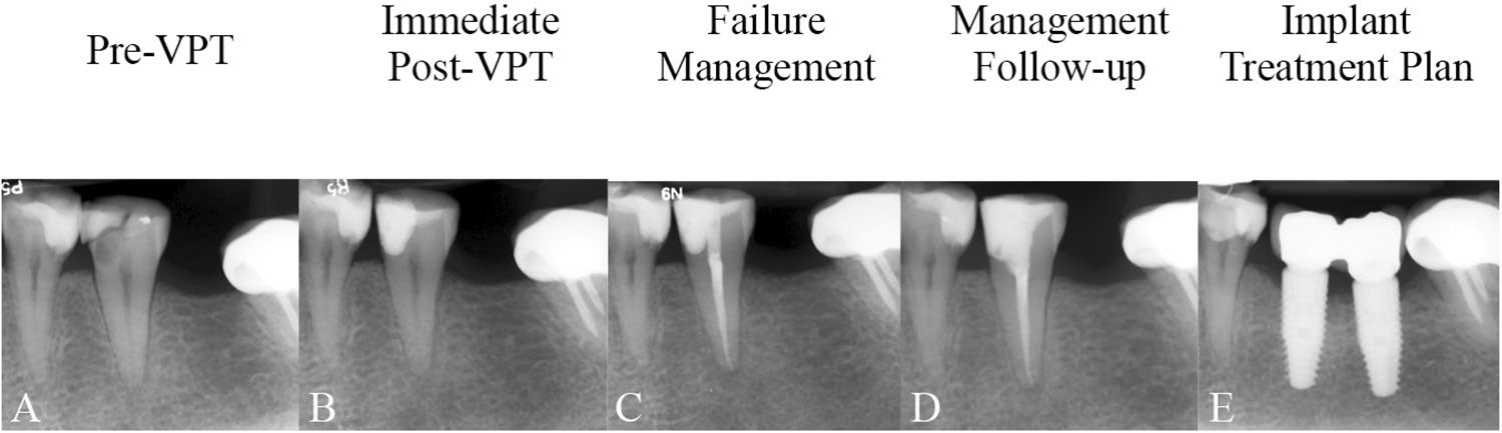
(**A**) Recurrent carious lesion on the left lower second premolar of a 58-year-old woman diagnosed with irreversible pulpitis/apical periodontitis; (**B**) Direct pulp capping and composite filling, which failed on 7-month follow-up due to symptomatic apical periodontitis; (**C**) Root canal treatment and resin composite filling of the tooth completed in a single session; (**D**) The tooth was functional and asymptomatic at the 3-month follow-up; (**E**) At 45-month follow-up, the radiograph showed that the tooth had been extracted and replaced with an implant.

The success and survival rates of the management procedures were 91.78% and 95.79%, respectively; the endpoint event observed was tooth extraction after RCT in four cases. These rates reflect the outcomes of the second retreatment modality for initial VPT failures, following an observation period ranging from ≤ 1 month to 9 years. The loss of teeth was because of fractures, periodontal disease progression, or prosthetic/implant treatment plans (Fig. 7).

### Survival time and comparisons

**Figure 8:**
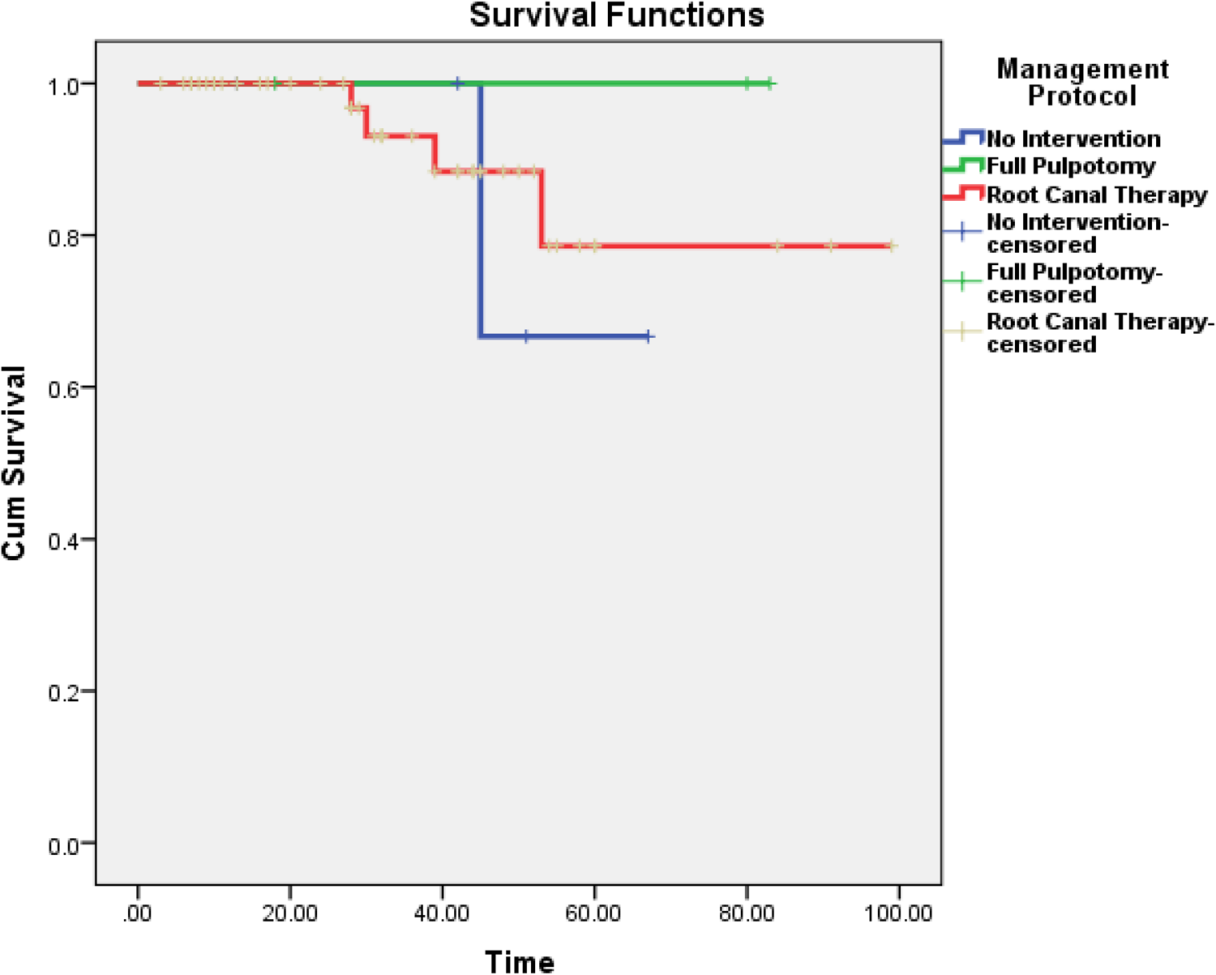
The Kaplan–Meyer survival curve produced for the VPT failed cases grouped by management protocol.

Table 3 shows the mean survival time (months) based on the management protocol. The Kaplan–Meyer survival curves for the management protocol are shown in Fig. 8 and the Log-Rank test showed no statistical difference (P = 0.704).

**Table 3:** The mean of survival time (months) based on the management protocol.

Management protocol	Estimated mean	Standard error	Lower limit for 95% CI	Upper limit for 95% CI
RCT	34.74	3.26	28.18	41.31
FP	60.33	21.18	0.00	151.48
NI	43.60	8.78	19.21	67.99
Overall	36.94	3.14	30.64	43.24

*CI* confidence interval.

## Discussion

The study aimed to assess 105 cases of unsuccessful outcomes within a larger cohort of 1257 VPT-treated teeth; with the original study reporting an overall success rate of 91.6%^18^. The study did not find evidence to reject the null hypothesis, indicating that these failures occurred throughout ≤ 1–108 months after initial VPT. In failed VPT cases, teeth with symptomatic IP and those with AP or widened PDL were significantly more common. Additionally, the majority of failures occurred in teeth with composite resin restorations. The observed success rates of the applied management strategies (i.e. RCT or re-VPT), assessed over an average follow-up of ~ 37 months, were ~ 92%, with a survival rate of ~ 96%. These outcomes align closely with reported success and survival rates for the original study^18^. Furthermore, the study’s exploration of diverse management protocols adds significant practical value. The presented range of approaches, including RCT, further re-VPT with more extensive pulp tissue penetration/removal, no new intervention for asymptomatic cases, and tooth extraction, offers clinicians a comprehensive toolkit when addressing failed VPT cases. This versatility in management options allows practitioners to tailor interventions based on individual patient characteristics, preferences, and the specific nature of the VPT failure encountered.

The majority of VPT failures excluding those with no symptoms/discomfort and including those retreated with another VPT, underwent RCT, resulting in remarkably high success and survival rates. It is noteworthy that the endodontist encountered partial calcification of root canals in only one case during RCT, where the canals’ orifices were successfully identified without magnification. Although the occurrence of pulp canal calcification (PCC) after VPT is recognized as a potential complication^21^, significant calcifications are not expected to occur within a short observation period. While some researchers/clinicians suggest that retreatment of a failed pulpotomy may be perceived as an easier procedure than managing a tooth with filled root canal treatments^22^, the current evidence on the incidence, magnitude, and influential factors of PCC in VPT-treated teeth remains insufficient^21^. Importantly, even if calcification occurs in root canals after VPT, it tends to manifest as canal narrowing rather than complete obliteration, making it generally manageable considering the advancements in endodontic techniques^22^.

In this investigation, teeth with more extensive caries (i.e. at least three lost surfaces) comprised over half of the unsuccessful VPT cases. While it may seem that the remaining tooth directly impacts the success or failure of VPT procedures, as emphasized in a randomized clinical trial^13^, it is essential to recognize that this is often indicative of underlying issues such as deep caries lesions or fractures rather than solely the structural integrity of the tooth itself.

Notably, a majority of teeth diagnosed with VPT failure in this study lacked radiographic signs of dentinal bridge formation, a factor recognized for its potential to safeguard pulp and ensure long-term tooth preservation^23,24^. However, it is essential to acknowledge the challenges encountered by some researchers in identifying dentinal bridges in the majority of VPT cases^13^.

The ultimate loss of teeth in this study was attributed to non-endodontic reasons, including fracture, improper crown/root ratio due to periodontal disease progression, or prosthetic/implant treatment plans. This observation aligns with a study that indicated the extraction of endodontically treated teeth due to non-endodontic reasons substantially impacted their overall survival rate, citing reasons such as recurrent caries, vertical root fractures, and periodontal disease progression^25^.

A therapeutic strategy explored for managing teeth with failed VPT involved a conservative approach, entailing the application of re-VPT with more extensive pulp tissue penetration/removal. However, a relatively limited number of teeth underwent this approach in our study. Among these cases, some demonstrated complete success upon re-VPT, as evidenced by favorable outcomes^20^.

Systemic conditions among patients with failed VPT-treated teeth were examined, revealing ~ 3% with type 2 diabetes and ~ 10% with cardiovascular problems, including hypertension and coronary artery disease. Hypertension may affect bone healing processes^26^, while long-term corticosteroid therapy (~ 4%) and smoking status influence treatment outcomes^27^, warranting further investigation.

Hypothyroidism emerged as a prevalent systemic condition among patients experiencing failed VPT, with a prevalence higher than that observed in the general population^28^. Approximately 10.5% of individuals with failed VPT were diagnosed with overt hypothyroidism and were undergoing levothyroxine treatment. While this finding suggests a potential association between hypothyroidism and VPT failure, caution is warranted in interpretation. Our study did not conduct specific statistical analysis for hypothyroidism cases, and the number of identified cases may not be sufficient to draw definitive conclusions. However, it is noteworthy that hypothyroidism can disrupt bone turnover, affecting crucial processes like osteoblastic bone formation, osteoclastic bone resorption, and secondary mineralization^29^. Levothyroxine therapy, used to replace thyroid hormones, may further impact bone density and healing processes^30^. Consequently, such patients may experience delays in bone lesion healing.

The study’s strength lies in its comprehensive analysis of failed VPT cases in mature permanent teeth. It employs a rigorous methodology, including a large-scale cohort design, thorough data collection, and appropriate statistical analysis. While this study offers significant insights, it is essential to acknowledge its limitations. Firstly, the retrospective design inherently introduces biases and limitations associated with retrospective data collection. Secondly, the study relies on data from a single clinic, which may limit the generalizability of the findings to broader populations. Additionally, the sample size, although considerable, may still restrict the statistical power for detecting more nuanced associations. Despite these limitations, this research provides valuable insights into the complex reasons underlying VPT failures in mature permanent teeth.

## Conclusion

Our study underscores significant success and survival rates achieved through two management protocols (RCT and re-VPT) for failed VPT cases. The prevalence of hypothyroidism among patients with unsuccessful VPT highlights the importance of comprehensive patient assessments. Furthermore, the observation that nearly half of the failed cases were asymptomatic, emphasizes the need for meticulous diagnostics. These insights offer valuable guidance for clinicians, facilitating a deeper understanding of conservative treatments and their outcomes in managing unsuccessful VPT cases.

## Data Availability

The data that support the findings of the current study are available from the corresponding author upon reasonable request.
